# Management of lithium dosing around delivery: An observational study

**DOI:** 10.1111/bdi.12955

**Published:** 2020-06-30

**Authors:** Nina M. Molenaar, Eline M. P. Poels, Thalia Robakis, Richard Wesseloo, Veerle Bergink

**Affiliations:** ^1^ Department of Psychiatry Icahn School of Medicine at Mount Sinai New York City New York USA; ^2^ Department of Psychiatry Erasmus Medical Center Rotterdam The Netherlands; ^3^ Department of Psychiatry GGZ Delftland Delft The Netherlands; ^4^ Department of Obstetrics, Gynecology and Reproductive Science Icahn School of Medicine at Mount Sinai New York City New York USA

**Keywords:** delivery, infant, lithium, neonate, postpartum, pregnancy

## Abstract

**Objectives:**

Recommendations on lithium dosing around delivery vary, with several guidelines suggesting that lithium should be discontinued prior to delivery. We aimed to evaluate the validity of these recommendations by investigating 1) maternal lithium blood level changes following delivery, and 2) the association between neonatal lithium blood levels at delivery and neonatal outcomes.

**Methods:**

In this retrospective observational cohort study, we included women with at least one lithium blood level measurement during the final week of pregnancy and the first postpartum week. For aim 2, we included a subcohort of women with neonates for whom neonatal lithium blood levels (obtained from the umbilical cord or a neonatal vein puncture within 24 hours of delivery) were available.

**Results:**

There were a total of 233 maternal lithium blood level measurements; 55 (23.6%) in the week before delivery and 178 (76.4%) in the week after. There was no association between time and lithium blood level/dose ratio (Pearson correlation coefficient −0.03, *P* = .63). Additionally, we included a total of 29 neonates for whom a lithium measurement was performed within 24 hours postpartum. Maternal and neonatal lithium blood levels were strongly correlated. We observed no associations between neonatal lithium blood levels at delivery and neonatal outcomes.

**Conclusion:**

Based on our findings, we do not recommend lowering the dosage or discontinuation of lithium prior to delivery. Stable dosing can prevent subtherapeutic lithium serum levels, which is especially important in the postpartum period when relapse risks are highest.

## 1 INTRODUCTION

Women with bipolar disorder are at high risk of relapse in the postpartum period.^1^, ^2^ Especially women without prophylactic pharmacotherapy are at elevated risk of postpartum relapse, with a reported pooled prevalence rate of 66%.^1^ Effective treatment with pharmacotherapy is therefore of critical importance. Lithium is an effective mood stabilizer and is widely used as a first‐line treatment for bipolar disorder.^3^ Some women choose to start lithium prophylaxis immediately after delivery, but for other women, continuation of lithium during pregnancy is the best option, despite associated risks.^4^ Lithium use during the first trimester of pregnancy is associated with a dose dependent increased risk of congenital malformations.^5^, ^6^ An increased risk could not be found for lithium use during the second and third trimester.

Dosing of lithium can be challenging as a result of normal physiological adaptations of renal function throughout pregnancy.^7^ Lithium blood levels decrease gradually in the first and second trimester, returning to their preconception level in the third trimester.^8^, ^9^ As a consequence, there is a risk of subtherapeutic lithium levels in the first and second trimester, which might lead to an increase in the dose by clinicians. This, in turn, could lead to an increased risk of lithium intoxication in the third trimester and the postpartum period. Frequent monitoring of lithium blood levels during pregnancy is therefore recommended and dosage should be adjusted in order to remain within the therapeutic window (0.5 mmol/L to 1.2 mmol/L).^4^, ^8^, ^10^, ^11^

Several reviews and guidelines have provided clinical advice on dosing strategy during pregnancy and the postpartum, including strategies for dosing around delivery to minimize the risk of both maternal and neonatal complications. Some suggest dose reduction by 30%‐50% upon first signs of labor or immediately after delivery,^9^, ^12^, ^13^, ^14^, ^15^ and others recommend to stop lithium prior to delivery.^16^, ^17^, ^18^ The underlying rationale is two‐fold: 1) blood lithium levels may rise due to a decrease in lithium clearance and vascular volume following delivery, and 2) a previous study found an association between lithium blood levels around delivery and neonatal complications, suggesting that a lower dosage could reduce the complication rate.^19^

In the current study we aimed to evaluate the validity of the recommendations around delivery by further investigating maternal lithium blood level changes following delivery (aim 1) and by examining the association between neonatal lithium blood levels at delivery and neonatal outcomes (aim 2).

## 2 PATIENTS AND METHODS

This retrospective observational cohort study was part of a larger study for which all women referred to the psychiatric and obstetric out‐patient clinics of Erasmus Medical Center and Leiden University Medical Center between January 2003 and May 2018 were eligible.^8^ Women were included in the current study if they used lithium during pregnancy and at least one lithium blood level measurement was obtained during the final week of pregnancy and the first postpartum week (aim 1). From the medical records, we extracted demographic, psychiatric and obstetric data, lithium blood level measurements, daily lithium dose, dosing alterations, and the dosing frequency. For aim 2, we included a subcohort of women with neonates for whom neonatal lithium blood levels were available. Clinical protocols in the Erasmus Medical Center recommend clinical observation of all lithium exposed neonates during the first 5 days after birth. Neonatal lithium blood levels were obtained from the umbilical cord or a neonatal vein puncture within 24 hours of delivery. From the medical records, we extracted information on neonatal outcomes and complications, including mild and transient complications. Extracted neonatal outcomes included: preterm birth, birth weight, Apgar scores, cord blood pH‐values, cord blood Base Excess values, and admission to medium/high care. We extracted information of all reported complications, ranging from mild to severe, and categorized them by organ system: respiratory, circulatory, hematological, gastro‐intestinal, metabolic, neurological, and immune system (infections).

The study was approved by the medical ethical review board of Erasmus University Medical Centre (MEC‐2013‐319).

### 2.1 Statistical analysis

For aim 1, we calculated the lithium blood level/dose ratio for each measurement, and visualized (scatterplot) and tested (R‐squared) the correlation between time (−7 to +7 days of delivery date) and lithium blood level/dose ratio. Lithium citrate (Litarex 564mg = 6mmol lithium) dosages were multiplied by 0.395 in order to obtain lithium carbonate prescription equivalents (400 mg = 10.8 mmol lithium).

For our second aim, we first visualized (scatterplot) and tested (R‐squared) the correlation between maternal and neonatal lithium blood levels surrounding delivery. Sensitivity analyses (two sample t‐test and Mann‐Whitney U test) were used to assess whether mean neonatal blood levels differed between umbilical cord and neonatal vein puncture measurements. We then used linear and binary logistic univariate regression to examine the association between neonatal lithium blood levels and neonatal outcome measures (preterm birth, birthweight, Apgar scores, cord blood pH‐ and BE‐values, admission to medium/high care, and neonatal complications). No multivariate regression analysis was performed due to the limited number of pregnancies included. The Statistical Package for Social Sciences (SPSS) version 25.0 was used for data analyses and the significance level was set at 0.05, two sided.

## 3 RESULTS

### 3.1 Lithium blood level changes following delivery (Aim 1)

We identified 78 women with a total of 100 pregnancies who were referred to the specialized out‐patient university clinics of Rotterdam (n = 57) and Leiden (n = 21). The most common psychiatric diagnosis was bipolar spectrum disorder (n = 68, 87.2%), while the remaining women (n = 10, 12.8%) were diagnosed with schizoaffective disorder, depressive disorder, or borderline personality traits. Median parity of all pregnancies was 1 (range 0‐6) and mean age at delivery 34.6 years (SD 4.3).

**FIGURE 1:**
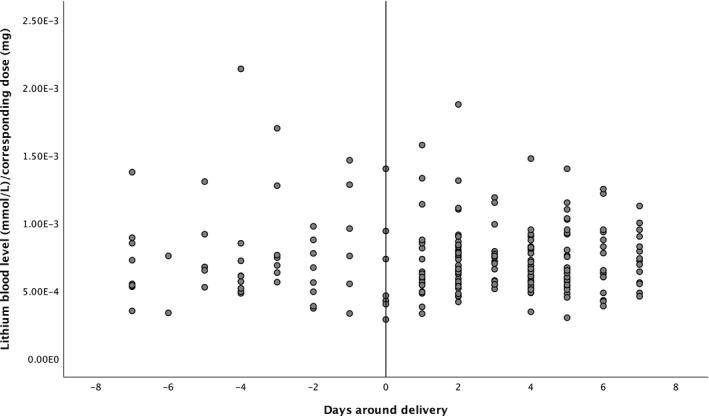
Course of lithium blood level/dose ration around delivery

There were a total of 233 lithium blood level measurements: 55 (23.6%) in the week before delivery and 178 (76.4%) in the week after. Mean lithium dosage was 1071 mg (SD 368) in the week before delivery and 1016mg (SD 284) in the week after delivery. Mean lithium blood level was 0.73 mmol/L in the week before delivery and 0.70 mmol/L in the week after delivery. The course of the lithium blood level/dose ratio before and after delivery can be seen in Figure 1. There was no association between time and ratio (Pearson correlation coefficient −0.03, *P* = .63). Lithium blood levels not normalized to dose can be found in Supplementary Figure 1.

### 3.2 Lithium blood levels and neonatal complications (Aim 2)

We included a total of 29 neonates for which a lithium measurement was performed within 24 hours postpartum (20 umbilical cord, 9 neonatal vein puncture). Cohort characteristics are represented in Table 1. While approximately half of the neonates had a complication, the majority of reported neonatal complications were mild and transient. One term neonate with fetal distress had complications in all seven organ systems, while having a neonatal lithium blood level of 0.72 mmol/L, and a birth weight of 4360 grams. A full overview of complications per neonate with additional lithium blood level can be found in Supplementary Table 1. All neonates with medium/high care admission were discharged in good medical condition, except for one neonate that was transferred to another hospital for further recovery from a respiratory infection.

**TABLE 1:** Maternal and neonatal characteristics of the sub cohort (aim 2)

Maternal characteristics	All (N = 29)
Lithium dosage in mg/day, mean (SD)^a^	1142.82 (350.74)
Lithium blood level in mmol/L, mean (SD)	0.67 (0.23)
Complications during delivery, n (%)^b^	16 (55.2)
Neonatal characteristics	
Lithium blood level in mmol/L, mean (SD)	0.61 (0.31)
Preterm (<37 weeks), n (%)	3 (10.3)
Birth weight in grams, mean (SD)	3589.14 (457.16)
Apgar score 1 minute, median (IQR)	8 (2)
Apgar score 5 minutes, median (IQR)	9 (2)
pH‐value cord blood, mean (SD)	7.24 (0.10)
Base Excess value cord blood, mean (SD)	−4.50 (5.12)
Admission medium/high care, n (%)	13 (44.8)
Duration admission medium/high care in days, median (IQR)	3 (4)
Any complication (including mild/transient), n (%)^c^	14 (48.3)
Neurological complications, n (%)	5 (17.2)
Respiratory complications, n (%)	5 (17.2)
Circulatory complications, n (%)	1 (3.4)
Gastro‐intestinal complications, n (%)	1 (3.4)
Infectious complications, n (%)	4 (13.8)
Hematological complications, n (%)	1 (3.4)
Metabolic complications, n (%)	7 (24.1)

^a^ Lithium dosage closest to delivery.

^b^ Observed complications: fetal distress (n = 7), postpartum hemorrhage (n = 5), prolonged rupture of the membranes (n = 5), increased duration second stage of labor (n = 3), preterm birth (n = 3), shoulder dystocia (n = 1), retained placenta (n = 1), meconium amniotic fluid (n = 1).

^c^ Details of complications: neurological – hypotonia (n = 3), tremors (n = 1), irritability (n = 1); respiratory – asphyxia with no spontaneous breathing after birth (n = 1), dyspnea (n = 1), cyanosis (n = 1), decreased oxygen saturation due to vomiting (n = 1), impaired breathing coordination (n = 1); circulatory – bradycardia (n = 1); gastro‐intestinal – cholestasis (n = 1); infectious – pneumonia (n = 1), observation/treatment for suspected infection (n = 3); hematological – disseminated intravascular coagulation (n = 1); metabolic – hyperbilirubinemia (n = 6), transient abnormal thyroid levels (n = 1).

**FIGURE 2:**
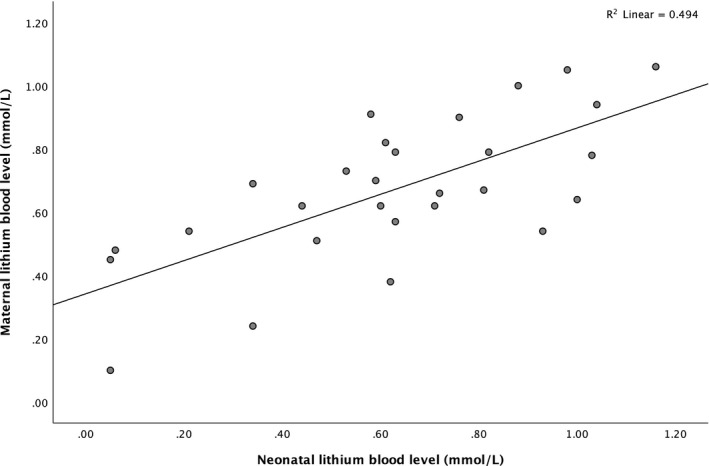
Correlation between maternal and neonatal lithium blood levels around delivery. Maternal lithium blood levels were obtained between 2 days prior to delivery and 6 days after delivery. Neonatal blood levels were obtained from the umbilical cord (n = 20) or neonatal vein puncture within 24 hours after delivery (n = 9)

There was a strong positive correlation between maternal and neonatal lithium blood levels (Pearson correlation coefficient 0.703, *P* < .001), which is visualized in Figure 2. Sensitivity analyses showed no significant difference in mean neonatal blood levels between umbilical cord and neonatal vein puncture measurements (two sample t‐test, *P* = .288; Mann‐Whitney U test, *P* = .390).

Univariate linear and logistic regression analysis showed no associations between neonatal lithium blood levels and complications during delivery (B = 11.8, 95% CI 0.8;181.1, *P* = .1), preterm birth (B = 8.2, 95% CI 0.1;746.7, *P* = .4), birth weight (B = 79.8, 95% CI −496.1;655.7, *P* = .8), Apgar score at 1 minute (B=−1.2, 95% CI −3.9;1.4, *P* = .4) and 5 minutes (B = −0.8, 95% CI −2.8;1.1, *P* = .4), cord blood pH‐value (B = −0.1, 95% CI −0.2;0.0, *P* = .2), cord blood BE‐value (B = −6.2, 95% CI −12.7;0.4, *P* = .1), admission to medium/high care (B = 1.8, 95% CI 0.2;20.1, *P* = .6), and neonatal complications (B = 1.2, 95% CI 0.1;12.9, *P* = .9).

## 4 DISCUSSION

In this retrospective observational cohort study, we found no maternal lithium blood level fluctuations surrounding delivery. Maternal and neonatal lithium blood levels were strongly correlated. We observed no association between neonatal lithium blood levels at delivery and neonatal outcomes.

Several guidelines recommend, out of caution, lowering or discontinuing lithium prior to labor to avoid high plasma lithium levels.^13^, ^14^, ^15^, ^16^, ^17^, ^18^ Blood levels are assumed to rise due to a decrease in lithium clearance and contraction of fluid volume following delivery, possibly reaching toxic levels. These recommendations are primarily based on reviews and case studies rather than on observational data of the target population, as cohort studies are sparse due to methodological difficulties. Our data indicates that lithium plasma levels do not increase during labor after correcting for the prescribed lithium dose.

A second argument for decreasing or discontinuing lithium treatment just before labor is the belief that a lower neonatal lithium blood level at time of delivery reduces the risks of lithium side‐effects in the neonate. This argument is based on the important study by Newport et al,^19^ in which lithium concentrations and obstetrical outcomes were available for 10 neonates, plus for another 14 neonates identified from published reports. Infants were grouped into a low and high lithium exposure group (cut‐off of 0.64 meq/L). They found that the high lithium exposure group had a higher rate of complications compared to the low lithium group, including central nervous system and neuromuscular complications, longer duration of infant hospital stay, and lower 1‐minute Apgar scores. In our sample of 29 neonates, we did not find a significant association between neonatal lithium blood levels and neonatal outcomes. A potential explanation for these contrasting findings is that neonatal blood level range differed substantially between our sample and the sample of Newport et al.^19^ The high lithium exposure group in Newport's study was predominantly composed of the neonates from previous case reports, who often had a lithium blood level higher than 0.7 and with some neonates classified as being within the toxic range (>1.2 mmol/L). Their low lithium exposure group existed mainly of women who had suspended their lithium treatment before delivery, and lithium levels were mostly subtherapeutic (<0.5 mmol/L). In our sample, most women were within the therapeutic window and no toxic levels were observed. Neonatal lithium levels might be associated with neonatal complication rate only if high (toxic) lithium dosages are used. Moreover, in the Newport paper, the overall complication rate of 100% in the high exposure group was driven by case reports on this topic for which publication bias is likely. Case studies are in origin a tool to disseminate information on unusual clinical syndromes, disease associations, or unusual side effects to therapy,^20^ and therefore in this case more likely to be published if neonatal complications were present with high lithium levels.

The high rate of neonatal complications (48.3%) in our study sample should be interpreted keeping in mind that lithium use during pregnancy is an indication for neonatal observation during the first five days following birth. Due to this observation period and the knowledge of lithium exposure during pregnancy, several mild complications might have been detected and recorded that otherwise would have gone unnoticed. Fortunately, even though the rate of complications was high, most complications were mild and transient.

This study is not without limitations. Statistical power was limited for examining the association between neonatal lithium levels and neonatal outcomes, even though we report on the largest sample thus far. In addition, neonatal lithium levels are not routinely assessed in clinical settings. Selective sampling might have contributed to relatively high neonatal lithium levels, as well as to a high complication rate.

Lithium dosing during pregnancy can be challenging due to changes in clearance throughout the trimesters. Relapse risk during pregnancy is not elevated and some authors even suggest that pregnancy is protective for relapse.^21^ Lithium levels in the lower range are often accepted, especially during the first trimester, in which there is a dose dependent increased risk for congenital malformations.^22^ In general, we recommend to monitor lithium levels frequently until 34 weeks of pregnancy, for example once every three weeks, followed by weekly monitoring until delivery. Lithium levels should not exceed therapeutic levels during pregnancy, as this may cause harm to the pregnant woman and her developing child. Based on the results of this study, we do not recommend to lower the dosage or discontinue lithium prior to delivery when lithium is used within the therapeutic window, unless this is warranted by special circumstances such as severe dehydration or renal dysfunction. Lowering the lithium dosage prior to delivery could lead to a subtherapeutic blood level and, as a consequence, insufficient protection against maternal relapse in the postpartum period, when relapse risks are highest.^23^ Instead, we recommend to carefully monitor lithium blood levels around delivery, and secure adequate fluid management. After delivery, we recommend lithium blood levels be obtained once at day 2 postpartum, followed again by (bi‐)weekly monitoring, and dosage adjustments when necessary. A high target therapeutic lithium blood level (eg 0.8‐1.0 mmol/L) immediately after delivery and during the first month postpartum is recommended to optimize relapse prevention.

## 5 DECLARATIONS

*Ethics approval and consent to participate:* The study was approved by the medical ethical review board of Erasmus University Medical Centre (MEC‐2013‐319). Due to the retrospective nature of the study, the need for consent was waived.

## Supporting information

Supplementary Material

## Data Availability

The data that support the findings of this study are available on request from the corresponding author. The data are not publicly available as they contain information that could compromise research participant privacy.
