# Supplementary material for: Management of lithium dosing around delivery: An observational study
Source: Bipolar Disord. 2020 Jun 30;23(1):49–54. doi: 10.1111/bdi.12955 (PMC7891390; doi:10.1111/bdi.12955)

**Supplementary Table 1.** Neonatal complications per infant

| **Nr.** | **Lithium blood level (mmol/L)** | **Complications** |
| --- | --- | --- |
| 1. | 0.05 | Hypotonia |
| 2. | 0.05 | Impaired breathing coordination |
| 3. | 0.06 | Observation/treatment for suspected infection |
| 4. | 0.47 | Observation/treatment for suspected infection |
| 5. | 0.58 | Hyperbilirubinemia, irritability |
| 6. | 0.62 | Cyanosis |
| 7. | 0.63 | Transient abnormal thyroid levels |
| 8. | 0.63 | Hyperbilirubinemia |
| 9. | 0.71 | Hypotonia, hyperbilirubinemia |
| 10. | 0.72 | Hypotonia, hyperbilirubinemia, bradycardia, cholestasis, asphyxia with no spontaneous breathing after birth, disseminated intravascular coagulation, pneumonia |
| 11. | 0.98 | Hyperbilirubinemia |
| 12. | 1.00 | Decreased oxygen saturation due to vomiting |
| 13. | 1.04 | Hyperbilirubinemia, dyspnea, observation/treatment for suspected infection |
| 14. | 1.16 | Tremors |

**Supplementary Figure 1**. Maternal lithium blood levels around delivery not normalized to dose


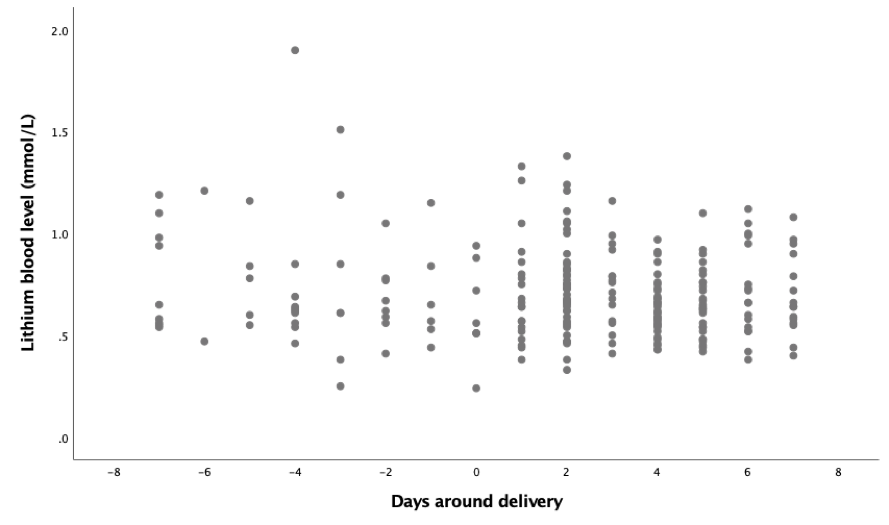

Supplement: Supplementary file 1 — Supplementary Material [file BDI-23-49-s001.docx]
